# Molecular prevalence and genotypic distribution of human pegivirus-1 among Iranian hemodialysis patients

**DOI:** 10.1016/j.virusres.2025.199582

**Published:** 2025-05-07

**Authors:** Amin Naseri, Enayat Anvari, SeyyedehMasumeh Mirnurollahi, Abolfazl Fateh

**Affiliations:** a Department of Biology, CTC, Islamic Azad University, Tehran, , Iran; b Department of Physiology, School of Medicine, Ilam University of Medical Science, Ilam, Iran; c Department of Mycobacteriology and Pulmonary Research, Pasteur Institute of Iran, Tehran, Iran; d Microbiology Research Center (MRC), Pasteur Institute of Iran, Tehran, Iran

**Keywords:** HPgV-1, Hemodialysis, Genotype 2a, Co-infection, Iran

## Abstract

This study investigates the molecular prevalence and genotypic distribution of Human Pegivirus-1 (HPgV-1) in Iranian hemodialysis (HD) patients. A case-control study was conducted from May 2017 to December 2024, including 1576 HD patients and 1000 age- and gender-matched healthy individuals. Serum samples were analyzed using nested PCR and sequencing of the 5′-UTR region to detect HPgV-1 RNA and determine genotypes. The prevalence of HPgV-1 was significantly higher in HD patients (13.6 %) compared to healthy controls (0.6 %). Among HPgV-1-positive HD patients, only genotype 2a was identified. Co-infections were notable, with 11.8 % of HPgV-1-positive patients also infected with HCV (predominantly genotype 3a), 3.0 % with HBV, and 11.7 % with HIV. Interestingly, HCV co-infected patients exhibited lower liver enzyme levels, while those co-infected with HIV had significantly higher CD4+ *T* cell counts (605.2 ± 198.7 vs. 412.3 ± 156.8 cells/mm³, *P* < 0.001), suggesting potential immunomodulatory effects of HPgV-1. Additionally, factors such as prolonged dialysis duration, elevated urea levels, and older age were significantly associated with HPgV-1 positivity. These findings underscore HD as a major risk factor for HPgV-1 transmission, likely exacerbated by hospital-acquired practices in dialysis units. The observed associations between HPgV-1 and improved clinical parameters in co-infected individuals highlight its complex role in viral pathogenesis, warranting further investigation into its underlying mechanisms. This study emphasizes the urgent need for stringent infection control measures in dialysis settings to mitigate viral transmission.

## Highlights

• The prevalence of HPgV-1 was significantly higher in HD patients (13.6 %) compared to healthy controls (0.6 %).
• Among HPgV-1-positive HD patients, only genotype 2a was identified.
• Co-infections were notable, with 11.8 % of HPgV-1-positive patients also infected with HCV (predominantly genotype 3a), 3.0 % with HBV, and 11.7 % with HIV.
• HCV co-infected patients exhibited lower liver enzyme levels, while those co-infected with HIV had significantly higher CD4+ *T* cell counts.

## 1 Introduction

The incidence of chronic kidney disease (CKD) is increasing globally. In recent years, the prevalence and incidence of end-stage renal disease (ESRD) have notably increased in Iran. However, compared to developed Western countries, the incidence of ESRD in Iran remains lower (Mousavi et al., 2014). Additionally, given the significant number of kidney transplants in Iran, it is fortunate that the prevalence of patients undergoing hemodialysis (HD) is not as high as in other regions globally (Haghighi et al., 2008). By the end of 2016, approximately 29,200 individuals were receiving HD treatment in Iran. The distribution of HD patients across various provinces at that time ranged from 225 to over 450 individuals per million inhabitants (Ahmadi et al., 2021).

Among HD patients, infectious complications are the second leading cause of morbidity and mortality (Collins et al., 2010). Infection with viral hepatitis, particularly hepatitis C virus (HCV), a blood-borne viral infection, is notably common among patients undergoing HD (Timofte et al., 2020). Patients undergoing maintenance HD and kidney transplantation are more frequently exposed to parenterally transmitted viruses such as HPgV-1, which—though not associated with clinical disease—can serve as indicators of transmission dynamics and co-infection patterns in this vulnerable population. Although HPgV-1 is primarily transmitted through blood transfusions, other routes of transmission may also contribute. Among HD patients, the prevalence of HPgV-1 viremia is relatively high and appears to correlate with both the number of transfusions received and the duration of dialysis treatment (De Lamballerie et al., 1996; Fabrizi et al., 1997; Masuko et al., 1996).

HPgV-1, previously known as GB virus C (GBV-C) or hepatitis G virus (HGV) was identified in 1996 during research on non-A-E hepatitis (Linnen et al., 1996). The virus has since been the subject of numerous studies evaluating its prevalence and potential pathogenicity (Stapleton, 2022; Yu et al., 2022). HPgV-1 infection can occur independently or alongside other infections such as HBV, HCV, or human immunodeficiency virus (HIV) due to shared transmission pathways. The virus is frequently identified in individuals with acute or chronic non-A-E hepatitis and has also been associated with hepatitis-related aplastic anaemia. However, it can also be present in healthy individuals without any known disease (Yu et al., 2022).

The central question concerns the potential pathogenic role of HPgV-1 in individuals undergoing HD. Consistent evidence suggests that the virus does not play a significant role in liver disease. Multiple studies have shown that liver enzyme levels remain within normal ranges in HD patients infected with HPgV-1 alone, indicating no signs of liver dysfunction. These findings further support the conclusion that HPgV-1 has limited pathogenic potential (Kao et al., 1999; Masuko et al., 1996; Zhu et al., 2003).

Although the risk of bloodborne virus transmission in HD settings is well documented, data on HPgV-1—a parenterally transmitted, non-pathogenic virus—remains scarce in Iranian HD populations. Assessing HPgV-1 prevalence and genotype distribution in this group not only contributes to regional epidemiological knowledge but may also provide insights into co-infection dynamics with HCV, HBV, and HIV and help monitor viral transmission within dialysis units using HPgV-1 as a surrogate marker. To address this, an epidemiological study was conducted to determine the prevalence of HPgV-1 infection and the distribution of its genotypes in HD patients.

## 2 Material and methods

### 2.1 Study population

**Fig. 1:**
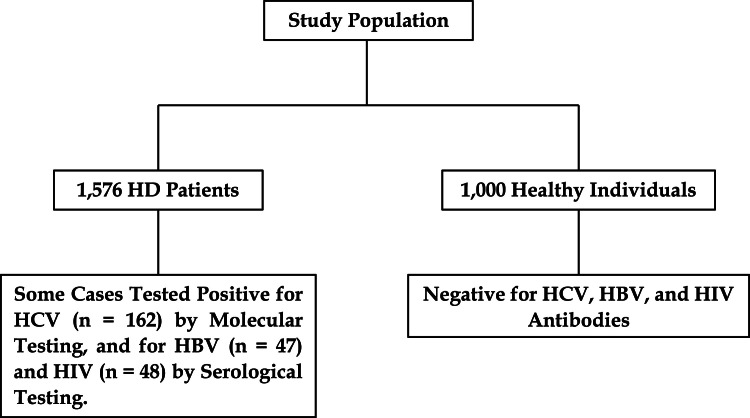
Flow Diagram Illustrating Study Participant Selection. HD, hemodialysis; HCV, hepatitis C virus; HBV, hepatitis B virus; HIV, human immunodeficiency virus.

This case-control study included 1576 HD patients and 1000 healthy individuals matched by age and gender, conducted from May 2017 to December 2024. All serum samples were tested for anti-HCV antibodies, HBsAg, and HIV antibodies using the enzyme-linked immunosorbent assay (ELISA) kit (Radim SpA, Pomezia, Rome, Italy) following the manufacturer’s instructions (Fig. 1).

This study followed the principles outlined in the Declaration of Helsinki (1975) and complied with local regulations. It also received approval from the Ethics Committee of the Islamic Azad University, Central Tehran Branch, in Tehran, Iran (IR.IAU.CTB.REC.1404.008). All participants were fully informed about the study procedures and experiments, and written informed consent was obtained from each individual before enrollment.

Data on liver enzymes profile, fasting blood glucose (FBS), lipid profiles, uric acid, serum urea, direct bilirubin, total bilirubin, creatinine, phosphorus, calcium, sodium, potassium, C-reactive protein (CRP), 25-hydroxyvitamin D, hemoglobin, white blood cells (WBC), and erythrocyte sedimentation rate (ESR) along with the duration of hemodialysis, were collected from patient records.

### 2.2 Extraction of RNA and cDNA synthesis

A 10-mL blood sample was collected from each patient and transferred into an EDTA-containing tube. Viral RNA was then extracted using the AccuPrep® viral RNA extraction kit (Bioneer Corp., Daejeon, South Korea) following the manufacturer’s guidelines. Complementary DNA (cDNA) synthesis was performed using the Transcriptor First Strand cDNA Synthesis Kit (Roche Diagnostics GmbH, Mannheim, Germany) in accordance with the manufacturer’s instructions.

### 2.3 Detection and genotyping of HCV

A nested PCR method targeting the 5′ non-coding region was performed to detect HCV using four specific oligonucleotide primers. In the first round of PCR (264 bp), the primers HCV-251F (5′-AGCGTCTAGCCATGGCGT-3′) and HCV-22R (5′-GCACGGTCTACGAGACCT-3′) were utilized, while in the second round (174 bp), HCV-FN-F2 (5′-GTGGTCTGCGGAACCGG-3′) and HCV-RN-R2 (5′-GGGCACTCGCAAGCACCC-3′) were employed. The first round of PCR was carried out under the following conditions: initial denaturation at 95 °C for 5 min, followed by 30 cycles of 94 °C for 45 s, 59 °C for 45 s, and 72 °C for 45 s, with a final extension at 72 °C for 5 min. The second round followed the same protocol, except for an increased annealing temperature of 62 °C. Additionally, a PCR-restriction fragment length polymorphism assay was used to determine HCV genotypes, which were further confirmed through sequencing of the 5´ non-coding region fragments (Pohjanpelto et al., 1996).

### 2.4 Analysis of HPgV-1 RNA and sequencing

Nested PCR was performed using four primers designed from the 5′ untranslated region (5′-UTR) of HPgV-1 for RNA amplification (Handajani et al., 2000). In the first amplification step, 5 μL of cDNA sample was amplified in a 12.5-μL of PCR master mix (SinaClon, Iran) and 0.3 μL of two outer primers: forward (5′-GCCAAAAGGTGGTGGATGGG-3′) and reverse (5′-CGGAGCTGGGTGGCCCCATGC-3′). Five μL of the first-round PCR product (376 bp) was used to a second round of PCR using nested primers: sense (5′-TGGTAGGTCGTAAATCCCGG-3′) and antisense (5′-TGGTCCTTGTCAACTCGCCG-3′). Both PCR rounds followed the same cycling conditions: an initial denaturation at 94 °C for 4 min, followed by 35 cycles of denaturation at 94 °C for 1 min, annealing at 51 °C for 45 s, and extension at 72 °C for 45 s. The second-round PCR amplified a 262 bp fragment.

The sequencing of the nested-PCR products was conducted by Bioneer Company in South Korea. BLAST was used to determine the genotypes of all samples by comparing them with online sequences. The phylogenetic tree was constructed using the bootstrapping method in MEGA 5 software (Biodesign Institute, USA).

### 2.5 Statistical analysis

Data analysis was performed using SPSS version 24.0 (2016; IBM Corp., Armonk, NY, USA). The normality of continuous variables was evaluated using the Shapiro-Wilk test. Pearson's Chi-square test was used for categorical variables, while the Mann-Whitney U test was applied to assess continuous variables. A *P*-value of <0.05 (two-tailed) was considered statistically significant.

## 3 Results

### 3.1 HD patients' baseline features

The present study involved 1576 patients undergoing HD and 1000 healthy individuals. Table 1 provides an overview of the demographic characteristics, clinical features, and laboratory parameters of both groups. In summary, the mean age of HD patients was 45.3 ± 10.8 years, while that of healthy individuals was 44.8 ± 9.6 years. The number of males among HD patients and healthy individuals was 1059 (67.2 %) and 665 (66.5 %), respectively. The duration of HD in patients was 4.9 ± 2.6 years.

**Table 1:** Baseline and Biochemical Information in Hemodialysis Patients and Healthy Subjects.

Variables	Hemodialysis Patients (*n* = 1576)	Healthy Subjects (*n* = 1000)
Mean age ± SD	45.3 ± 10.8	44.8 ± 9.6
Gender (male/female)	1059/517 (67.2/32.8 %)	665/335 (66.5/33.5 %)
ALT, IU/L (mean ± SD) (Reference range: 5–40)	36.4 ± 15.2	26.3 ± 11.3
AST, IU/L (mean ± SD) (Reference range: 5–40)	33.2 ± 17.8	26.7 ± 10.1
ALK, IU/L (mean ± SD) (Reference range: up to 306)	173.2 ± 136.2	162.3 ± 129.2
Cholesterol, mg/dL (mean ± SD) (Reference range: 50–200)	197.8 ± 49.8	118.1 ± 27.6
TG, mg/dL (mean ± SD) (Reference range: 60–165)	164.1 ± 43.6	131.7 ± 39.9
LDL, mg/dL (mean ± SD) (Reference range: up to 150)	147.8 ± 30.6	69.9 ± 18.7
HDL, mg/dL (mean ± SD) (Reference range: >40)	31.7 ± 12.8	34.2 ± 12.3
WBC, 10^9^/L (mean ± SD) (Reference range: 4000–10,000)	8203.4 ± 3134.8	7327.8 ± 2698.6
RBC, ×10^6^/µL (mean ± SD) (Reference range: 4.2–6.2)	3.6 ± 0.6	3.4 ± 0.4
ESR, mm/1st h (mean ± SD) (Reference range: 0–15)	19.7 ± 10.1	12.4 ± 7.6
FBS, mg/dL (mean ± SD) (Reference range: 70–100)	102.1 ± 34.1	89.8 ± 28.4
Platelets × 1000/cumm (mean ± SD) (Reference range: 140,000–400,000)	141 ± 81	192 ± 93
Urea, mg/dL (mean ± SD) (Reference range: 15–45)	112.4 ± 11.2	38.2 ± 9.8
Creatinine, mg/dL (mean ± SD) (Reference range: 0.6–1.4)	6.2 ± 1.1	0.8 ± 0.4
Uric acid, mg/dL (mean ± SD) (Reference range: 2.5–7.7)	6.9 ± 1.2	3.1 ± 0.9
Total bilirubin, mg/dL (mean ± SD) (Reference range: 0.2–1.2)	2.8 ± 1.6	0.7 ± 0.5
Direct bilirubin, mg/dL (mean ± SD) (Reference range: 0–0.2)	0.6 ± 0.2	0.1 ± 0.0
Hemoglobin, g/dL (mean ± SD) (Reference range: 12–18)	10.5 ± 1.6	13.2 ± 1.9
Sodium, mEq/L (mean ± SD) (Reference range: 134–148)	139.2 ± 2.1	141.5 ± 1.9
Potassium, mEq/L (mean ± SD) (Reference range: 3.5–5.3)	4.6 ± 0.7	5.1 ± 1.0
Calcium, mg/dL (mean ± SD) (Reference range: 8.6–10.3)	9.4 ± 0.8	9.6 ± 0.7
Phosphorus, mg/dL (mean ± SD) (Reference range: 2.6–4.5)	5.1 ± 1.1	3.9 ± 0.8
CRP, mg/L (mean ± SD) (Reference range: <10 mg/L Negative)	8.3 ± 5.9	5.2 ± 3.7
25-hydroxyvitamin D, ng/mL (mean ± SD) (Sufficiency: 21–150)	21.4 ± 5.2	41.6 ± 13.6
Duration of hemodialysis (years) (mean ± SD)	4.9 ± 2.6	–
HCV genotypes (*n* = 162, 10.3 %)		
1a	46 (28.4 %)	–
1b	43 (26.5 %)	–
3a	73 (45.1 %)	–
HBV	47 (3.0 %)	–
HIV	48 (3.0 %)	–
HPgV-1	214 (13.6 %)	6 (0.6 %)

ALT, alanine aminotransferase; AST, aspartate aminotransferase; ALP, alkaline phosphatase; TG, triglyceride; LDL, low density lipoprotein; HDL, high density lipoprotein; WBC, white blood cells; RBC, red blood cells; ESR, erythrocyte sedimentation rate; FBS, fasting blood glucose; CRP, C-reactive protein; HCV, hepatitis C virus; HBV, hepatitis B virus; HIV, human immunodeficiency virus; HPgV-1, human pegivirus 1; SD, standard deviation. *Statistically significant (< 0.05).

Of the 1576 HD patients, 162 (10.3 %) were infected with HCV, with genotype distribution as follows: 46 (28.4 %) had genotype 1a, 43 (26.5 %) had genotype 1b, and 73 (45.1 %) had genotype 3a. Additionally, 47 (3.0 %) of HD patients were infected with HBV, while 48 (3.0 %) tested positive for HIV using the ELISA test.

### 3.2 Baseline features of HD patients with HPgV-1 infection

The prevalence of HPgV-1 was 214 (13.6 %) among HD patients and 6 (0.6 %) among healthy individuals. Table 2 provides a summary of the baseline demographic characteristics of HD-positive patients with HPgV-1. In HPgV-1 positive patients, the mean age (*P* = 0.029), urea (*P* = 0.038) and duration of HD (*P* = 0.019) were significantly higher compared to those without HPgV-1.

**Table 2:** Comparison Epidemiological and Laboratory Properties Between Hemodialysis Patients with and without HPgV-1 Infection.

Variables	Positive (*n* = 214)	Negative (*n* = 1362)	*P*-value
Mean age ± SD	45.5 ± 10.8	43.7 ± 10.9	0.029*
Gender (male/female)	149/65 (69.6/30.4 %)	910/452 (66.8/33.2 %)	0.434
ALT, IU/L (mean ± SD) (Reference range: 5–40)	38.9 ± 13.2	37.3 ± 11.9	0.150
AST, IU/L (mean ± SD) (Reference range: 5–40)	35.9 ± 10.7	34.8 ± 19.8	0.248
ALK, IU/L (mean ± SD) (Reference range: up to 306)	172.1 ± 129.1	169.2 ± 128.9	0.144
Cholesterol, mg/dL (mean ± SD) (Reference range: 50–200)	195.4 ± 47.9	198.1 ± 49.6	0.321
TG, mg/dL (mean ± SD) (Reference range: 60–165)	163.8 ± 42.9	164.7 ± 43.0	0.582
LDL, mg/dL (mean ± SD) (Reference range: up to 150)	148.2 ± 32.1	146.9 ± 33.0	0.087
HDL, mg/dL (mean ± SD) (Reference range: >40)	32.7 ± 12.8	32.2 ± 12.3	0.367
WBC, 10^9^/L (mean ± SD) (Reference range: 4000–10,000)	8123.4 ± 3224.8	8024.8 ± 2701.6	0.261
RBC, ×10^6^/µL (mean ± SD) (Reference range: 4.2–6.2)	3.5 ± 0.6	3.3 ± 0.5	0.421
ESR, mm/1st h (mean ± SD) (Reference range: 0–15)	18.8 ± 10.6	18.4 ± 9.9	0.098
FBS, mg/dL (mean ± SD) (Reference range: 70–100)	101.7 ± 33.5	100.9 ± 29.8	0.486
Platelets × 1000/cumm (mean ± SD) (Reference range: 140,000–400,000)	140 ± 81	139 ± 79	0.249
Urea, mg/dL (mean ± SD) (Reference range: 15–45)	114.4 ± 10.8	108.9 ± 10.0	0.038*
Creatinine, mg/dL (mean ± SD) (Reference range: 0.6–1.4)	6.1 ± 1.1	5.8 ± 0.9	0.329
Uric acid, mg/dL (mean ± SD) (Reference range: 2.5–7.7)	6.8 ± 1.0	6.6 ± 0.8	0.189
Total bilirubin, mg/dL (mean ± SD) (Reference range: 0.2–1.2)	2.7 ± 1.5	2.5 ± 0.9	0.379
Direct bilirubin, mg/dL (mean ± SD) (Reference range: 0–0.2)	0.6 ± 0.3	0.5 ± 0.2	0.211
Hemoglobin, g/dL (mean ± SD) (Reference range: 12–18)	10.1 ± 1.3	9.2 ± 0.8	0.087
Sodium, mEq/L (mean ± SD) (Reference range: 134–148)	140.1 ± 1.6	139.8 ± 1.1	0.354
Potassium, mEq/L (mean ± SD) (Reference range: 3.5–5.3)	4.8 ± 0.9	4.1 ± 0.8	0.178
Calcium, mg/dL (mean ± SD) (Reference range: 8.6–10.3)	9.6 ± 1.0	9.5 ± 0.8	0.092
Phosphorus, mg/dL (mean ± SD) (Reference range: 2.6–4.5)	5.2 ± 1.3	4.9 ± 0.9	0.628
CRP, mg/L (mean ± SD) (Reference range: <10 mg/L Negative)	7.6 ± 5.1	7.9 ± 4.7	0.526
25-hydroxyvitamin D, ng/mL (mean ± SD) (Sufficiency: 21–150)	22.1 ± 4.9	21.6 ± 6.6	0.621
Duration of hemodialysis (years) (mean ± SD)	5.5 ± 3.2	4.8 ± 2.5	0.019*
HCV genotypes (*n* = 162, 10.3 %)			<0.001*
1a	24 (11.2 %)	22 (1.6 %)	
1b	16 (7.5 %)	27 (2.0 %)	
3a	14 (6.5 %)	59 (4.3 %)	
HBV	10 (3.0 %)	37 (2.7 %)	0.094
HIV	25 (11.7 %)	23 (1.7 %)	<0.001*

ALT, alanine aminotransferase; AST, aspartate aminotransferase; ALP, alkaline phosphatase; TG, triglyceride; LDL, low density lipoprotein; HDL, high density lipoprotein; WBC, white blood cells; RBC, red blood cells; ESR, erythrocyte sedimentation rate; FBS, fasting blood glucose; CRP, C-reactive protein; HCV, hepatitis C virus; HBV, hepatitis B virus; HIV, human immunodeficiency virus; HPgV-1, human pegivirus 1; SD, standard deviation.

⁎ Statistically significant (< 0.05).

Among 214 HPgV-1 positive patients, 24 (11.2 %), 16 (7.5 %), and 14 (6.5 %) were infected with HCV-1a, HCV-1b, and HCV-3a, respectively, with these differences being statistically significant (*P* < 0.001). The levels of ALT (32.3 ± 11.7 vs. 36.3 ± 12.8, *P* = 0.002), AST (31.9 ± 12.1 vs. 36.8 ± 13.1, *P* = 0.001), and ALK (164.2 ± 127.3 vs. 174.4 ± 130.2, *P* < 0.001) in HCV-positive patients co-infected with HPgV-1 were significantly lower compared to those without HPgV-1 infection.

Additionally, 10 (3.0 %) and 25 (11.7 %) of the HPgV-1 positive patients were co-infected with HBV and HIV, respectively, with the co-infection with HIV showing statistical significance (*P* < 0.001). The average CD4+ cell counts in HPgV-1 positive patients (605.2 ± 198.7 cells/mm^3^) were significantly higher than in those who were HPgV-1 negative (412.3 ± 156.8 cells/mm^3^) (*P* < 0.001).

### 3.3 Phylogenetic analysis

**Fig. 2:**
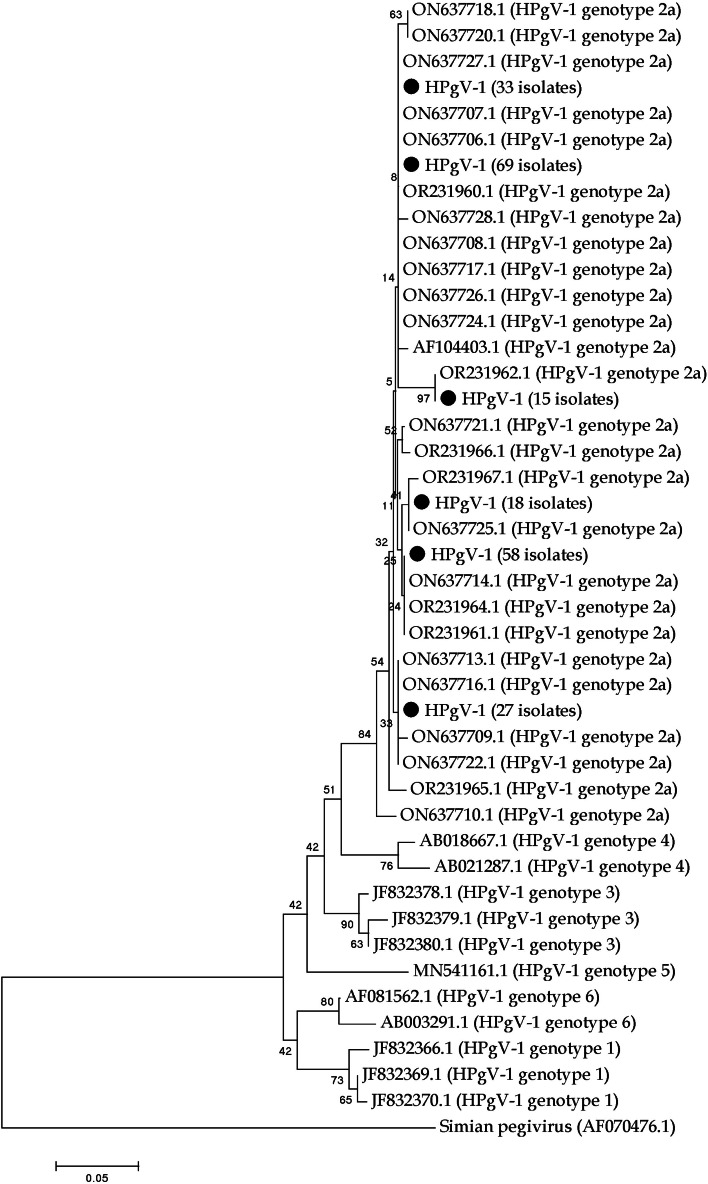
Phylogenetic analysis of HPgV-1 in hemodialysis patients.

The phylogenetic tree was generated using the bootstrapping method with MEGA 5 software, displaying six genotypes of HPgV-1, with genotype 2a being the most prevalent in Iran. The sequencing results of the 5′-UTR revealed that all positive samples were identified as genotype 2a, with co-infection of HCV, HBV and HIV present (Fig. 2).

## 4 Discussion

This study reports an HPgV-1 positivity rate of 13.6 % in HD patients and 0.6 % in healthy individuals, aligning with prevalence rates observed in other studies from Iran, which range from 5 % to 43 % (Ghanbari et al., 2010; Zali et al., 1999). Studies on HD patients, such as those by Salehi et al. (Salehi et al., 2014) and Dadmanesh et al. (Dadmanesh et al., 2015), show varying prevalence rates, with higher infection rates in HD patients due to factors like blood transfusions, nosocomial transmission, and gender or dialysis session frequency. A study in Ahvaz (2018) found 10 % of HD patients were infected, highlighting the importance of infection control (Sanchooli et al., 2018). Global studies show greater HPgV-1 prevalence in developing countries, particularly in at-risk groups, with rates ranging from 0.8 % to 46.6 % in healthy blood donors and 1.8 % to 75.3 % in high-risk individuals. This higher prevalence in poorer regions is linked to socioeconomic factors such as unregulated blood donations and unsafe medical practices (Yu et al., 2022).

Multiple Iranian studies have documented a notable rate of HPgV-1 and HCV co-infection, with Ghanbari et al. reporting 43.6 % (Ghanbari et al., 2010), Zali et al. 40 % (Zali et al., 1999), and Amini et al. 25 % (Amini et al., 2005), all higher than the rate observed in our study. This co-infection is attributed to overlapping transmission routes. Broader studies show that 11.8 % to 37.2 % of HCV-infected individuals also carry HPgV-1 (Samadi et al., 2022; Yu et al., 2022). Interestingly, our findings—along with prior research—suggest that HPgV-1 may exert a protective effect, as co-infected patients exhibited lower liver enzyme levels. Persistent HPgV-1 infection appears to mitigate liver damage in HCV-infected patients by modulating immune responses. Specifically, it results in reduced AST and ALT levels through the down-regulation of genes critical to intra-hepatic T-cell signal transduction (Feng et al., 2014). These genes—LCK, DOK2, IL2R-γ, and CCND3—are closely linked to the T-cell receptor (TCR) complex. Their suppression contributes to decreased liver inflammation and injury, thereby lessening the severity of chronic hepatitis C and reducing the risk of hepatopathy. This supports growing evidence that HPgV-1 may play a protective immunomodulatory role in HCV co-infection (Berzsenyi et al., 2011; Yu et al., 2022).

In this study, 3.0 % of HPgV-1 positive patients were co-infected with HBV, a rate that aligns with findings from other regions. While research on HPgV-1 and HBV co-infection is limited, existing studies show varying prevalence rates. For example, Javanmard et al. reported a 10 % co-infection rate, and Alvarado-Mora et al. found a similar 3.2 % rate in HBV-positive samples (Alvarado-Mora et al., 2011; Davod et al., 2013). Higher rates were observed in the UAE (14.3 %), Turkey (29 %), and Ghana (27 %), especially in individuals also infected with HIV (AbuOdeh et al., 2015; Akcali et al., 2006; N’Guessan et al., 2018). Despite the shared transmission routes of these viruses—such as blood exposure, sexual contact, and vertical transmission—current evidence suggests that HPgV-1 does not affect HBV disease progression or HBV-DNA levels (N’Guessan et al., 2018).

In this study, 11.7 % of HPgV-1 positive patients were co-infected with HIV, consistent with prior findings such as Ramezani et al.'s 10.97 % (Ramezani et al., 2008). Reported global co-infection rates range widely from 5 % to 47.9 % (de Pina-Araujo et al., 2021). Notably, HPgV-1 co-infection is associated with slower HIV progression and improved survival (Van Leth et al., 2005). This study also observed significantly higher CD4+ counts in HPgV-1 positive individuals, supporting evidence that HPgV-1 may exert a protective effect (Köksal et al., 2023; Liu et al., 2021). Mechanistically, HPgV-1 helps limit HIV-1 progression by interfering with viral entry and replication. It down-regulates HIV co-receptors CCR5 and CXCR4, while increasing their natural ligands, thereby hindering HIV cell entry. Additionally, HPgV-1′s E2 and NS5A proteins inhibit HIV-1 assembly by reducing viral structural proteins like P24 and P17. HPgV-1 also promotes immune homeostasis by stabilizing Th1/Th2 cytokine responses, which may further delay progression to AIDS. These findings suggest a beneficial immunomodulatory role for HPgV-1 in HIV-infected individuals (Bhattarai and Stapleton, 2012; Schwarze-Zander et al., 2010; Yu et al., 2022).

This study identified only genotype 2a of HPgV-1 among positive samples, consistent with previous findings across various Iranian cities, where genotype 2a has also been exclusively reported (Davod et al., 2013; Ghanbari et al., 2010). Globally, HPgV-1 genotypes show distinct geographic distributions: genotypes 1 and 2 are mainly found in Africa and Europe respectively, genotype 3 is prevalent in Asia and South America, genotypes 4 and 5 dominate in Southeast Asia, genotype 6 in Indonesia, and the newly discovered genotype 7 has been reported in Yunnan, China, and other Asian countries including Qatar. These geographic patterns reflect the virus's evolutionary history and transmission routes (Stapleton, 2022; Yu et al., 2022).

In conclusion, this study provides new regional data on the prevalence and genotype distribution of HPgV-1 among a large sample of Iranian HD patients. While the high prevalence highlights the known risks of viral exposure in dialysis settings, the observed associations between HPgV-1 infection and favorable co-infection markers—such as lower liver enzyme levels in HCV co-infection and higher CD4+ counts in HIV—suggest a potential immunomodulatory role for the virus that warrants further investigation. These findings may inform future research on HPgV-1 as a biomarker for co-infection outcomes and emphasize the importance of monitoring clinically benign viruses as indicators of transmission risk in healthcare environments.

## Ethical approval

This study followed the principles outlined in the Declaration of Helsinki (1975) and complied with local regulations. It also received approval from the Ethics Committee of the Islamic Azad University, Central Tehran Branch, in Tehran, Iran (IR.IAU.CTB.REC.1404.008). All participants were fully informed about the study procedures and experiments, and written informed consent was obtained from each individual before enrollment.

## Consent

All participants provided written informed consent.

## Funding

None.

## CRediT authorship contribution statement

**Amin Naseri:** Methodology, Investigation. **Enayat Anvari:** Validation, Formal analysis, Data curation. **SeyyedehMasumeh Mirnurollahi:** Writing – original draft, Supervision. **Abolfazl Fateh:** Writing – review & editing, Writing – original draft, Supervision.

## Declaration of competing interest

The authors declare that they have no known competing financial interests or personal relationships that could have appeared to influence the work reported in this paper.

## Data Availability

The data used to support the findings of this study are included within the article.
